# Antimicrobial, antibiofilm, and antiviral investigations using egyptian *phoenix dactylifera* L. pits extract

**DOI:** 10.1186/s13568-024-01695-3

**Published:** 2024-05-09

**Authors:** Hanaa H. Gomaa, Dalia Y. Amin, Alaaeldin R. Ahmed, Nader A. Ismail, Khaled A. El Dougdoug, Basma T. Abd-Elhalim

**Affiliations:** 1 Department of Botany and Microbiology, Faculty of Science, Suez Canal University, Ismailia, Egypt; 2 Department of Dermatology, Faculty of Medicine, Suez Canal University, Ismailia, Egypt; 3 Department of Agricultural Microbiology, Faculty of Agriculture, Ain Shams University, PO Box 68-Hadayek Shoubra, Shubra El-Khaimah, Cairo, 11241 Egypt

**Keywords:** Antimicrobial activity, Antibiofilm activity, Antiviral activity, Gas chromatography, *Phoenix dactylifera* L. pits

## Abstract

*Phoenix dactylifera L.* and its wastes are known to be high in nutrients that are beneficial to human health. The study aimed to evaluate the antimicrobial, antibiofilm, and antiviral properties of *Phoenix dactylifera* L. pits extract (PDPE) in vitro. Gas chromatography-mass spectrometry (GC–MS) analysis indicated phenol, 2,5-bis(1,1-dimethyl ethyl), tetradecanoic acid, octaethylene glycol monododecyl ether, á-D-glucopyranosiduronic acid, and heptaethylene glycol monododecyl ether existence. The PDPE influenced pathogenic microorganisms, with inhibition zone diameters (IZDs) ranging from 10.0 to 35.0 mm. *Staphylococcus aureus* ATCC 5638 had the highest IZD, while *Salmonella typhi* DSM 17058 and *Shigella sonnei* DSM 5570 had the lowest. The antifungal effect observed only in spore failure or conidia formation. PDPE showed a 100% antibacterial spectrum against bacteria, with MIC values between 250 and 1000 µg/ml. MIC was only indicated with *S. aureus* of 500 µg/ml. MBC values ranged from 500 to 1000 g/ml, with MBC values of 500 g/ml for *B. cereus*, *E. faecalis*, *S. typhi*, and *S. sonnei*. The activity was 66.7% at 500 µg/ml, further concentrations of 125–250 g/ml had no antibacterial effect. PDPE biofilm inhibition % had the highest percentage of inhibition (98.59%) with *S. aureus*, *B. cereus* (94.12%), and *E. coli* (74.46%). With 50% (CC_50_) viral activity, the highest non-toxic PDPE dose was found to be at 123.0 µg/ml.

## Key points

• *Phoenix dactylifera* L. pits extract (PDPE) has varied active compounds with different bioactivities.

• *Phoenix dactylifera* L. pits extract (PDPE) has a bactericidal mode of action for various pathogenic bacteria.

• *Phoenix dactylifera* L. pits extract (PDPE) has cytotoxicity 50% (CC_50_) value at 123.0 µg/ml with 50% viral activity.

## Introduction

In most nations, microbial infections are the primary cause of disease and mortality, accounting for 1.8 million cases of illness every year **(**Ahmad et al. 2022). Plant-derived natural goods and herbal medications are becoming increasingly popular as alternative remedies for various health issues in developing nations like Egypt. In this situation, the alternative medical approach should incorporate the screening, selection, and assessment of the pharmacological characteristics of naturally occurring phytochemicals derived from plants. Several fruits’ seeds, stones, and pits are utilized as supplementary medicine due to their phytochemical content, which aids in illness prevention, disease treatment, and the reduction of side effects and various forms of stress (El-Far et al. 2021). *P. dactylifera* pits’ functional food qualities in dietary treatment **(**Zihad et al. 2021), macro- and micronutrients **(**Ahmad et al. 2022), phenolic acids, as an ingredient in bread **(**Abdel-Shaheed et al. 2021), and protein solubility **(**Aljaloud et al. 2020) have all been documented in several studies. Egypt is recognized as one of the nations that produces *P. dactylifera*. A meaty pericarp and seed make up the *P. dactylifera* palm’s fruit **(**Mia et al. 2020). About 15% of the weight of the *P. dactylifera* fruits is made up of pits. Complementary and alternative medicine (CAM) uses the seeds of several fruits to prevent or lessen illness side effects and stress **(**Mirza et al. 2018).

*P. dactylifera* seed contains a variety of chemical components, including zinc (Zn), cadmium (Cd), calcium (Ca), and potassium (K), as well as unsaturated fatty acids like oleic and linoleic acids that may inhibit the 5-α reeducates enzymes **(**Mia et al. 2020). Moreover, seeds have substantial concentrations of dietary fiber (78–80 g/100 g), antioxidants, and phenolics (3102–4430 mg equivalents/100 g gallic acid) **(**Al Alawi et al. 2020). The possible health advantages of the seed extract for humans have been studied and it is also utilized in several traditional treatments **(**Gregorova et al. 2020). *P. dactylifera* seeds have also been shown to contain a significant amount of antioxidants **(**Saryono et al. 2019).

Furthermore, numerous studies show that *P. dactylifera* pits are a rich source of naturally occurring antioxidants rather than a waste product, and they may even be employed as antibacterial candidates to combat fungus and both positive Gram-negative (G-ve) and positive bacteria (G + ve) **(**Platat et al. 2019). Numerous pathogens, including *Salmonella* spp., *Campylobacter* spp., *Escherichia coli*, *S. aureus*, *L. monocytogenes*, *E. faecalis*, *S. agalactiae*, and *B. cereus*, have been shown to exhibit antimicrobial action **(**Daoud et al. 2019). Furthermore, it has been demonstrated that the phenolic content is highly dependent on the extraction solvent and is consistently higher in aqueous extracts than in alcoholic extracts **(**Daoud et al. 2019; Platat et al. 2019).

The main aim of this study is to determine the active compounds present in Egyptian *P. dactylifera* L. pits extract (PDPE). In addition, examine the impact of PDPE on viruses, fungi, and Gram-positive and Gram-negative bacterial pathogens. The significant findings could be utilized in future research to develop products that can use *P. dactylifera* pit extract in wound healing and as a food additive to get rid of biofilm and contamination issues.

## Materials and methods

### Chemicals, media, and reagents

Ethyl acetate, Nutrient Agar (CM0003B), Malt agar (CM0059), and Mueller Hinton agar (PO1191) were purchased from OXOID, UK. Standard antibiotics of Ampicillin, Streptomycin, and Fluconazole (1000 µg/ml) were purchased from Amoun Pharmaceutical Company, Cairo, Egypt. All chemicals are analytical grades.

### Collection of ***Phoenix dactylifera ***L. pits samples

Pits of *P. dactylifera* L. from Tamr El Wadi variety were obtained from New Valley, Egypt in November 2022 at the Tamr stage.

### Preparation of ***P. dactylifera***pits ethanolic extract (PDPE)

The *P. dactylifera* pits (PDP) were extracted, washed, dried, and ground into a fine powder using a Cutting Mill (SM 400, RETSCH, Germany). The PDP ethanolic extract were prepared according to Bouhlali et al. (2015) by weighting 100 g of *P. dactylifera* pits powder dissolved in 500 ml absolute ethanol and mixed well, then left for 24 h at ambient room temperature. Next, the mixture was filtered by filter paper (Whatman No.1) and concentrated by rotary vacuum evaporator at 40 °C then left to dry. Then collected and stored in dark-dry place for further investigations.

### Microbial strains collection source

Nine different strains of pathogens were tested in this study, comprising six types of bacteria and three types of fungi. These strains included *Bacillus cereus* ATCC 11778, *Staphylococcus aureus* ATCC 5638, *Enterococcus faecalis* ATCC 7080, *Salmonella typhi* DSM 17058, *E. coli* ATCC 8379, *Shigella sonnei* DSM 5570, *Aspergillus flavus* ATCC 9643, *Penicillium chrysogenum* ATCC 10106, and *Aspergillus niger* DSM 1957. All the strains were obtained from the Microbiology Department at the Faculty of Agriculture, Ain Shams University, Egypt. The pathogens were stored and maintained in nutrient and malt agar medium at 4 ºC and were cultured overnight at 37 ºC and 28 ºC in nutrient and malt broth medium for the antimicrobial activity tests (Galal et al. 2021).

### Standard inoculum preparation of microbial pathogen strains

The standard inoculum of the pathogens was made by following the protocol outlined in Abd-Elhalim et al. (2019). To prepare the inoculum, a loop of bacteria culture was taken from a newly prepared culture and placed into a 50 ml nutrient broth medium. The mixture was then shaken in a shaker incubator (Shin Saeng, South Korea) for 24 h at 37 °C, with the speed set at 150 rpm. For fungi, the spore suspension was inoculated onto malt broth media and incubated for 72 h at 28 °C, with the speed set at 150 rpm.

### Gas Chromatography-Mass Spectrometry (GC-Mass) analysis of PDPE active compounds

GC-MS analysis was performed at The Regional Center of Mycology and Biotechnology in Cairo, Egypt. Fifty grams of PDPE was soaked in 500 ml of Ethyl acetate - HLPC crude - C_4_H_8_O2 - absolute for 24 h. The extract was then concentrated under reduced pressure until it was dry, at a temperature no higher than 60 °C. According to Franchi et al. (1985), 0.2 g of PDPE was dissolved in 1 ml of HPLC grade ethyl acetate and then injected into a GC mass spectrometer (Thermo Scientific, USA). The temperature was first adjusted at 35 °C then increased gradually to reach 200 °C after 180 s. Then the temperature increased again to reach the final temperature at 280 °C. The injector temperature was adjusted at 250 °C and the transfer line temperatures kept at 260 °C, the carrier gas (helium) flow rate was at 1 ml/min. The solvent fellow was fixed for 3 min, then diluted samples (1 L) was injected with Autosampler (AS1300) in split mode. At 70 eV ionization voltages of m/z 40 to 1000 in full scan mode, the EI mass spectra were recorded, as the temperature of ion source was kept at 200 °C. The active compounds were identified through comparing the mass spectra and retention times to databases of WILEY 09 and NIST 11.

### Antimicrobial influence of PDPE

Antimicrobial activity was tested using the well-diffusion method, as described by Abd-Elhalim et al. (2019) and Galal et al. (2021). This involved creating wells in the Muller Hinton agar layer using a sterilized cork borer with a diameter of 9.0 mm. After planting 50 µl of standard microbial inoculum cultures (10^6^ CFU/ml) and evenly dispersing the medium in the petri dish, each sterile petri dish was filled with sterile Muller Hinton and malt agar for bacteria and fungi, respectively. Control PDPE (1000 µg/ml) and standard antibiotic were added to each well, and the wells were incubated for 24 h at 37 °C for bacteria and 72 h at 28 °C for fungi. The inhibitory zones were measured in millimeters and compared to the diameter of the standard reference antibiotic. The activity index (AI) was calculated using the formula described by Galal et al. (2021).

1 $$AI = {{Diameter\,of\,the\,inhibition\,zone\,by\,PDPE} \over {Diameter\,of\,the\,inhibition\,zone\,by\,the\,standard\,antibiotic}}$$

#### Evaluation of PDPE minimal inhibitory concentration (MIC)

The lowest concentration of an antimicrobial chemical or natural agent that stops a microorganism’s observable development is known as the minimum inhibitory concentration, or MIC. To verify microbial resistance to an antimicrobial agent and track the efficacy of novel antimicrobial agents, minimum inhibitory concentrations (MICs) are crucial in diagnostic laboratories. To calculate the MICs, the recommendations of the National Committee for Clinical Laboratory Standards (CLS) were used, as mentioned by Humphries et al. (2018). Two-fold serial dilutions (1/2, 1/4, and 1/8) of PDPE were made in sterilized water to achieve final concentrations of 1000 µg/ml (control), 500 µg/ml, 250 µg/ml, and 125 µg/ml. These dilutions were then transferred into wells made in inoculated plates, which were previously prepared. Using a calibrated micropipette, bacteria inocula, and fungi spore suspensions were prepared and added to MHA and malt agar welled plates. Then incubated at 37 °C and 28 °C for 24 and 72 h for bacteria and fungi, at the same arrangement.

#### Estimation of PDPE minimal lethal centration (MLC)

The minimal lethal concentration (MLC) for bacteria (MBC) or fungi (MFC) is the lowest amount of an antimicrobial agent that can stop the visible growth of microorganisms. To determine this concentration, the microorganisms are first exposed to varying concentrations of the agent, and then the growth from the minimal inhibitory concentration (MIC) stage is transferred to agar plates. These plates are then incubated at 37 °C for 24 h for bacteria and at 28 °C for 72 h for fungi. Any growth or lack thereof is then observed and recorded (Abd-Elhalim et al. 2019).

#### Effluence of PDPE

After obtaining the MIC and MLC, the MLC/MIC ratio was calculated. If the ratio is equal to or greater than 4, it indicates that the PDPE has a bactericidal effect. However, ratio values equal to 2 or less indicate a static action (Abd-Elhalim et al. 2019).

2 $$\displaylines{PDPE\,\,antimicrobial\,\,action \cr\,\,\,\,\,\,\,\,\,\,\,\,\,\,\,\,\,\,\,\,\,\,\,\,\,\,\,\,\,\,\,\,\,\,\,\,\,\,\,\,\,\,\,\,\,\,\,\,\,\,\,\,\,\,\,\,\,\,\,\,\,\,\,\,\,\,\,\,\,\,\,\,\,\,\,\, = {{Minimum\,\,lethal\,\,centration\,\,\left( {MLC} \right)} \over {Minimum\,\,inhibition\,\,concentration\,\,\left( {MIC} \right)}}}$$

### Antibiofilm activity of PDPE

After performing a test tube assay against selected pathogenic microbes, the results were compared with control non-treated samples, followed by a semi-qualitative assay to determine microbial biofilm hindrance **(**Elakraa et al. 2022). Prior to the antibiofilm assay, the pathogens inoculated in the tested bacteria were incubated overnight at 37 °C. For the antibiofilm test, 0.5 ml of liquid nutrient broth was mixed with the fixed microbes in designed test tubes and incubated overnight at 37 °C. Following incubation, all treated and untreated tubes were discarded, and all tubes were cleaned with phosphate buffer saline (PBS; pH 7.0) and washed several times with deionized water. The adhered microbial cells in the tubes were then fixed with 3.5% sodium acetate (5 ml) for about 15 min and finally cleaned several times with deionized water. The tubes with the fixed microbial biofilm were stained with 5 ml of 0.15% crystal violet (CV) for about 15 min to estimate the semi-qualitative antibiofilm activity of the samples. To determine the semi-quantitative antibiofilm potential of the samples, the CV-stained microbial cells were dissolved by the action of ethanol solution (5 mL), and the optical density (O.D.) of the dissolved CV was measured using the UV-visible spectrophotometry method at 570 nm. The microbial biofilm inhibition percentage was then estimated using the following equation **(**Ansari et al. 2014):

3 $$\left[ {{{(O.D.\,\,Control\,\, sample - O.D.\,\, treated\,\, sample)} \over {O.D.\,\,Control\,\, sample}}} \right] \times 100$$

### Antiviral effect of PDPE

Antiviral activity against Herpes simplex virus (HSV1) obtained from Vacsera company Agouza, Giza, was evaluated using the Petricevich and Mendonça (2003**)** method. The method involves comparing the virus titers in the presence and absence of test inactivates, and the difference between the two refers to the antiviral activity of the test inactivates. Vero cells were pretreated with the test inactivates for 24 h before being inoculated with the virus. The Vero cells were cultured in 96-well cell culture plates and counted at 10^5^ cells/ml. Once the cells reached confluency, the Eagle’s minimal essential growth medium (EMEM) was discarded. The plates were then incubated at 37 °C for 24 h with non-toxic concentrations (100 µl/well). Control plates were kept untreated during this time to facilitate viral control titration. The HSV 1 was then serially diluted ten times in the E-MEM medium. Each virus dilution was injected as 0.1 ml/well into each well after the growth medium was disposed of. Regardless of whether the Vero cell culture plates had been exposed to test inactivates or not, each virus dilution was injected. Untreated and uninfected wells were maintained as a negative control for cell culture. The plates were incubated at 37 °C and subjected to daily examinations using an inverted microscope. The endpoint of cytopathic effect (CPE) assay Reed (1999**)** was used to measure the viral titers in test material treated and untreated cells after 72 h. This endpoint is determined by counting the number of wells per dilution that demonstrated CPE. It is also referred to as the 50% cell culture infectious dose (CCID_50_). The 50% endpoint was calculated according to Reed and Muench (1938**)**.

4 $$\eqalign{& 50\% \, \, end\, \, point\, \, \left( {CCID50} \right) \cr & \, = {{(Percentile\, \, of\, \, CPE > 50\% - 50)} \over {(Percentaile\, \, of\, \, CPE > 50\% - Percentile\, \, of\, \, CPE < 50\% )}} \cr & \, \times \, \, Log\, \, dilution \cr}$$

All titrations were performed three times. The difference in mean viral titers between treated and untreated plates indicates antiviral activity.

### Statistical analysis

All collected data were analyzed using IBM® SPSS® Statistics software (2017) and a Duncan test with a P-value of 0.05 was conducted according to Duncan (1955**)**.

## Results

### Gas Chromatography-Mass Spectrometry (GC-Mass) analysis of PDPE

**Fig. 1:**
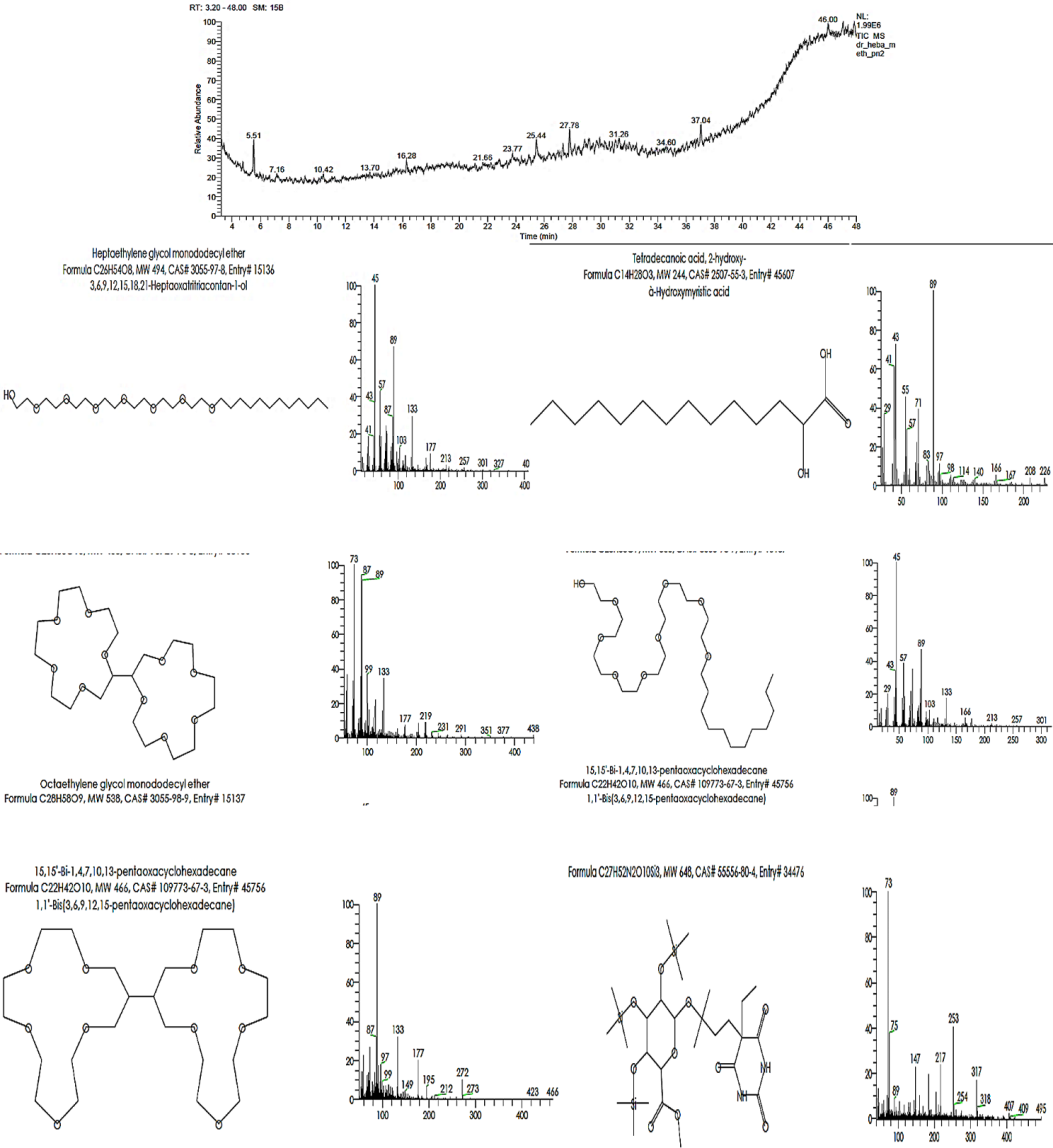
Chromatographic analysis of bioactive compounds in *P. dactylifera* L. pits ethanolic extract (PDPE)

Based on Table 1; Fig. 1, the GC-mass analysis determined the phytochemical composition of PDPE. It confirmed that the PDPE contains essential and effective components that exhibit numerous bioactive activities. These components as phenol, 2,5-bis(1,1-dimethyl ethyl), heptaethylene glycol monododecyl ether, tetradecanoic acid, octaethylene glycol monododecyl ether.

### Antimicrobial activity of PDPE

Table 2 indicates that PDPE was effective against all the tested pathogenic microorganisms, including fungi and bacteria. The inhibition zone diameters (IZDs) ranged from 10.0 to 35.0 mm on well-agar diffusion plates. The control standard antibiotics had IZDs ranging from 30.0 to 40.0 mm. Among all the microorganisms tested, *S. aureus* ATCC 5638 showed the most significant IZD of 35.0 mm and AI of 0.88, which is 0.14-fold lower than the standard antibiotic. However, both *S. typhi* DSM17058 and *S. sonnei* DSM 5570 had the lowest PDPE influence, with IZDs of 26.0 mm and AIs of 0.65 and 0.82, respectively. The fungal strains *A. flavus* ATCC9643, *P. chrysogenum* ATCC10106, and *A. niger* DSM 1957 did not show a zone of inhibition. Instead, there was a failure of spores and conidia formation for all the tested strains, as shown in Table 3.

**Table 1:** GC-MS analysis of the chemical composition (%) of *P. dactylifera* L. pits ethanolic extract (PDPE)

RT	Compound Name	Area %	M.W
27.78	Phenol, 2,5-bis(1,1-dimethyl ethyl)	49.41	C_14_H_22_O
5.52	Tetradecanoic acid, 2-hydroxy-	50.59	C_14_H_28_O_3_
5.52	(2 S,2’S)-2,2’-Bis[1,4,7,10,13-pentaoxacyclopen tadecane]	50.59	C_20_H_38_O_10_
5.52	Octaethylene glycol, mono-dodecyl ether	50.59	C_28_H_58_O_9_
5.52	15,15’-Bi-1,4,7,10,13-pentaoxacyclohexadecane	50.59	C_22_H_42_O_10_
27.78	á-D-Glucopyranosiduronic acid	49.41	C_27_H_52_N_2_O_10_Si_3_
27.78	2,4-Imidazolidinedione, 5-[3,4-bis[(trimethylsilyl)oxy]phenyl]-3-methyl- 5-phenyl-1-(trimethylsilyl)-	49.41	C_25_H_40_N_2_O_4_Si_3_
27.78	Cyclopenta[d]anthracene-8,11-dione, 1,2,3,3a,4,5,6,6a,7,8,11,12-dodecahydro-3-(1-m ethyl ethyl)-12-hydroxy-	49.41	C_20_H_26_O_3_
5.52	Heptaethylene glycol mono-dodecyl ether	50.59	C_26_H_54_O_8_
27.78	12,13-Dioxatricyclo[7.3.1.0(1,6)]tridecane, 10-methoxycarbonyl-5-(4-methylphenylsulfony loxy)-6-methyl-	49.41	C_21_H_28_O_7_S

*Rt = retention time, and M.W = Molecular Weight

**Table 2:** Inhibition zone diameter (IZD) of pathogenic bacteria strains impacts PDPE compared with control antibiotics after incubation at 37 °C for 24 h

Pathogenic strains (Bacteria)						
Inhibition zone diameter (IZD): mm						
PDPE concentration (µg/ml)	*B. cereus* ATCC 11778	*S. aureus* ATCC 5638	*E. faecalis *ATCC 7080	*S. typhi* DSM 17058	*E. coli* ATCC 8379	*S. sonnei* DSM 5570
**1000 (Control)**	29.0de ± 0.4	35.0b ± 0.03	30.0d ± 0.30	19.0gh ± 0.078	27.0e ± 0.33	26.0e ± 0.40
**500**	10.0j ± 0.15	34.0b ± 0.16	30.0d ± 0.11	18.0 h ± 0.098	27.0e ± 0.40	24.0ef ± 0.55
**250**	10.0j ± 0.30	32.0b, c ± 0.74	28.0de ± 0.43	13.0i ± 0.22	26.0e ± 0.44	23.0f ± 0.62
**125**	0.00	26.0e ± 0.040	26.0e ± 0.45	0.00	24.0ef ± 0.45	19.0gh ± 0.56
**Ab** **(1000 µg/ml)**	30.0^d^ ± 0.32	40.0^a^ ± 0.22	35.0^b^ ± 0.21	40.0a ± 0.25	33.0c ± 0.37	35.0b ± 0.29
**AI**	0.97	0.88	0.88	0.65	0.90	0.82

*Ab = standard antibiotics were Streptomycin and Ampicillin for G^+ ve^ bacteria and G^− ve^ bacteria, respectively. AI stands for activity index, mm stands for millimeter, and SE stands for standard error. According to Duncan (1955), variables in the same column that are distinguished by the same letter do not differ significantly

**Table 3:** Inhibition zone diameter (IZD) of pathogenic fungi strains with PDPE compared with a control antifungal after incubation at 28 °C for 72 h

Pathogenic strains (Fungi)			
Inhibition zone diameter (IZD) mm			
PDPE concentration (µg/ml)	*Aspergillus flavus* ATCC 9643	*Penicillium chrysogenum* ATCC 10106	*Aspergillus niger* DSM 1957
**1000 (Control)**	-	-	-
**500**	+	+	+
**250**	+	+	+
**125**	++	++	+
**Af** **(1000 µg/ml)**	13.0 ± 0.11	11.0 ± 0.27	11.0 ± 0.07

Af = Antifungal (Fluconazole 1000 µg/ml), +++= strong growth, ++= medium growth, += poor growth, and - = no growth

### Evaluation of MIC and MLC of PDPE

Table (4) results showed that PDPE had MIC values ranging from 250 to 1000 µg/ml against the tested pathogenic bacterial strains. Only PDPE with *S. aureus *ATCC 5638 was investigated at a MIC value of 500 µg/ml. All strains of *B. cereus* ATCC 11778, *E. faecalis* ATCC 7080, *S. typhi* DSM 17058, and *S. sonnei* DSM 5570 exhibited an MIC value of 250 µg/ml PDPE. However, only *the E. coli* ATCC 8379 strain showed an MIC value of 500 µg/ml. The results clearly demonstrate that PDPE has 100% antibacterial activity against the tested pathogenic strains at concentrations ranging from 500 to 1000 µg/ml, while at concentrations of 250 µg/ml, the activity was 83.3%. Additionally, a concentration of 125 µg/ml did not display any antibacterial activity against the tested pathogen strains. The MBC values of PDPE, as presented in Table, ranged from 500 to 1000 µg/ml against the tested pathogenic bacterial strains. Strains of *B. cereus* ATCC 11778, *E. faecalis* ATCC 7080, and *S. typhi* DSM17058 showed an MBC value of 500 µg/ml PDPE. Strains of *S. aureus* ATCC 5638, *S. sonnei* DSM 5570, and *E. coli* ATCC 8379 revealed an MBC value of 1000 µg/ml. The results clearly demonstrate that PDPE has 100% antibacterial activity against the tested pathogenic strains at a concentration of 1000 µg/ml, while at a concentration of 500 µg/ml, the activity was 66.7%. Concentrations ranging from 125 to 250 µg/ml did not exhibit any antibacterial influence against any of the pathogenic strains.

**Table 4:** Minimal inhibitory concentration (MIC) and Minimal lethal concentration (MLC) and spectrum activity of PDPE against pathogenic bacterial strains after incubation at 37 °C for 24 h

Pathogenic bacterial strains								
Minimal inhibitory concentration (MIC)								
PDPE (µg/ml)	*B. cereus* ATCC 11778	*S. aureus* ATCC 5638	*E. faecalis* ATCC 7080	*S. typhi* DSM 17058	*E. coli *ATCC 8379	*S. sonnei* DSM 5570	Spectrum Activity (%)	
**1000 (Control)**	-	-	-	-	-	-	6/6	100
**500**	-	-	-	-	-	-	6/6	100
**250**	-	-	-	-	+	-	5/6	83.3
**125**	+	+	+	+	+	+	0/6	0.00
**MIC value (µg/ml)**	250	250	250	250	500	250		
**Minimal bactericidal concentration (MBC)**								
**1000 (Control)**	-	-	-	-	-	-	6/6	100
**500**	-	-	-	-	+	+	4/6	66.7
**250**	+	+	+	+	+	+	0/6	0.00
**125**	+	+	+	+	+	+	0/6	0.00
**MBC value (µg/ml)**	500	1000	500	500	1000	1000		
**MBC/MIC Ratio**	2	2	2	2	2	4		
**Effect**	Bactericidal	Bactericidal	Bactericidal	Bactericidal	Bactericidal	Bacteriostatic		

- = No growth, + = growth. Results are averages of 3 replicates, Bactericidal = ≤ 2 and Bacteriostatic effect = ≥ 2

### Antibiofilm activity of PDPE

**Fig. 2:**
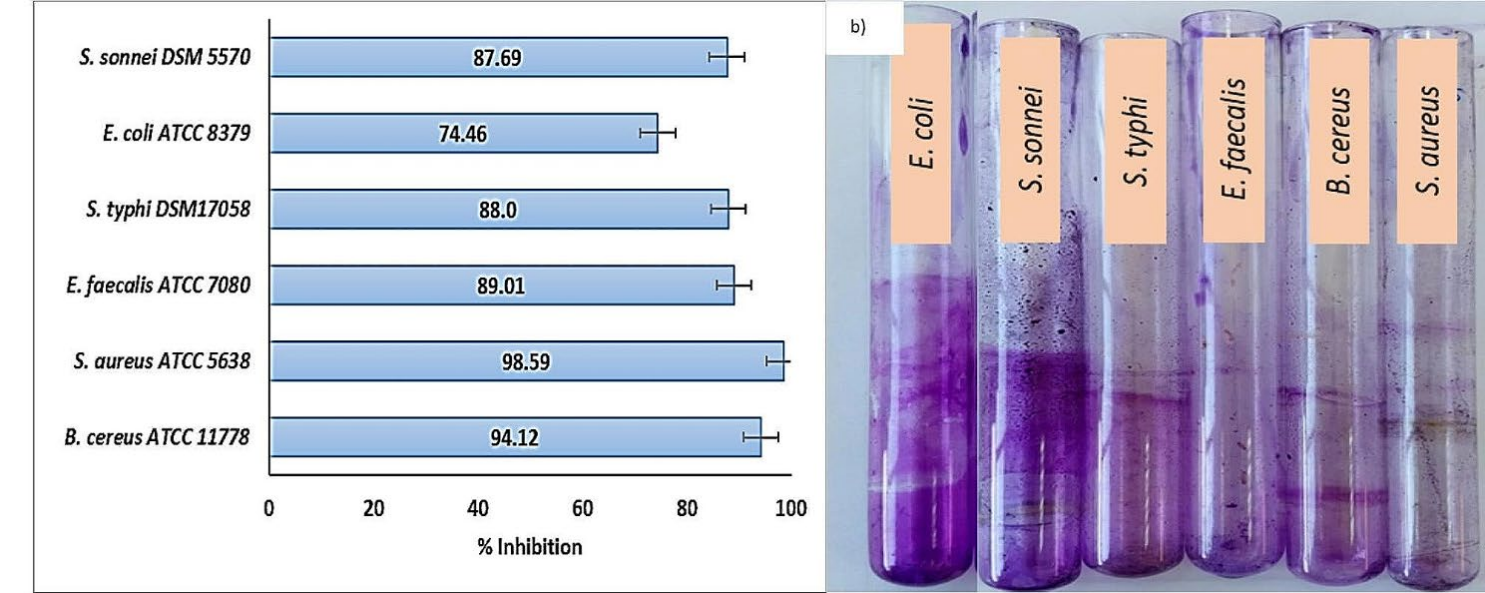
Antibiofilm efficiency of PDPE against different pathogenic strains **A**) Results recorded as % of inhibition, **B**) Tube method of antibiofilm activity pathogenic bacterial strains treated with PDPE

The percentage of biofilm inhibition was calculated and is shown in Table 5; Fig. 2 for PDPE. The highest inhibition percentage of PDPE (1000 µg/ml) was observed against *S. aureus* ATCC 5638, with a rate of 98.59%. *B. cereus* ATCC 11778 was the next most affected strain, with an inhibition rate of 94.12%. However, *E. coli* ATCC 8379 showed the lowest antibiofilm activity with a rate of 74.46%.

**Table 5:** Biofilm formation inhibition % for treated and non-treated pathogenic bacteria with PDPE

pathogenic bacterial strains	Crystal violet (CV) stain O.D. at 570 nm		Inhibition %
	Control	Treated with 1000 µg/ml PDPE	
*B. cereus* ATCC 11778	0.85^c^ ± 0.30	0.05^j^ ± 0.02	94.12
*S. aureus* ATCC 5638	0.71^e^ ± 0.25	0.01^k^ ± 0.11	98.59
*E. faecalis* ATCC 7080	0.91^b^ ± 0.80	0.10^h^ ± 0.08	89.01
*S. typhi* DSM 17058	0.75^d^ ± 0.49	0.09^h^ ± 0.08	88.00
*E. coli* ATCC 8379	0.94^a^ ± 0.62	0.24^g^ ± 0.12	74.46
*S. sonnei* DSM 5570	0.65^f^ ± 0.46	0.08^h, i^±0.44	87.69

Values are means ± SD (*n* = 3). Data within all groups are analyzed using ANOVA by Duncan’s test

### Antiviral activation of PDPE

**Fig. 3:**
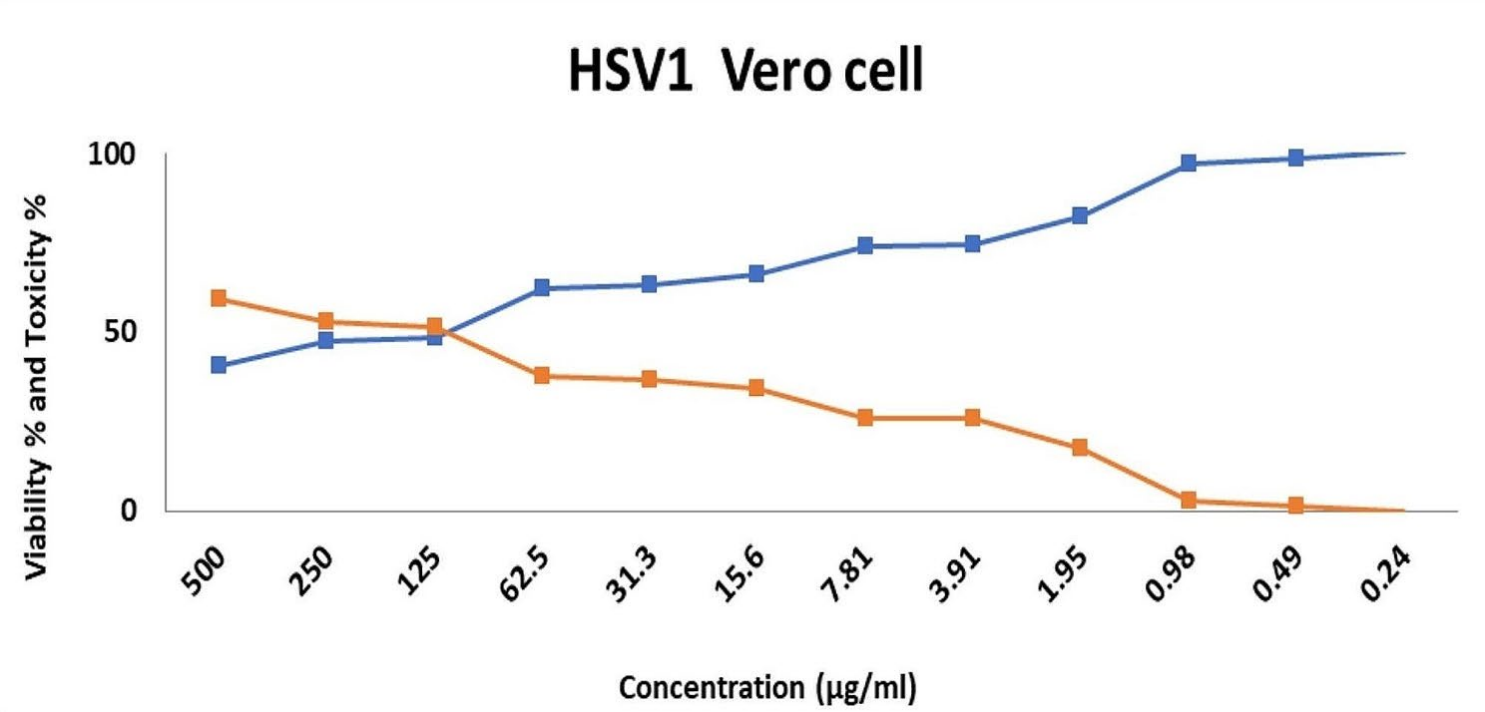
Line chart showing the effect of PDPE on both viability and toxicity of the tested cell lines Herpes simplex virus 1 (HSV1)

Table 6 presents the results of the viability and toxicity responses of the Herpes simplex virus (HSV1). In the first step, the maximum non-toxic concentrations of PDPE were determined. Figure 3 shows that the concentration of cytotoxicity 50% (CC_50_) values was 123.0 µg/ml, with 50% viral activity.

**Table 6:** Determination of maximum nontoxic concentration (MNTC) of PDPE on Vero cells; Herpes simplex virus 1 (HSV 1)

Concentration (µg/ml)	Viability %	Toxicity %	Concentration of cytotoxicity 50% (CC_50_) (µg/ml)
500	40.61	59.39	123.0
250	47.27	52.73	
125	48.48	51.52	
62.5	62.42	37.58	
31.3	63.33	36.67	
15.6	66.06	33.94	
7.81	73.94	26.06	
3.91	74.24	25.76	
1.95	82.42	17.58	
0.98	97.27	2.73	
0.49	98.79	1.21	
0.24	100.30	0.30	

## Discussion

*Phoenix dactylifera* L. fruit is a staple food in most Arabic-speaking countries. It’s composed of a soft, sweet pericarp covering a seed. Different parts of the *P. dactylifera* L palm tree can be used for medicinal purposes, such as dried leaves, fruit, pollen, seed, and tree bark extracts. A *P. dactylifera* L palm fruit consists of several components, including skin, pulp, endocarp, and seed **(**Shanableh and Radeef 2020**)**. There are over 5000 different types of *P. dactylifera* L worldwide, depending on their kind and maturation stage. *P. dactylifera* L palms contain various nutritive and cosmetic components, and these bioactive substances are used in business and medicine (El-Far et al. 2021).

Studies have shown that palm *P. dactylifera* L. have the potential to be antioxidants, antimutagenic, antibacterial, anti-inflammatory, antihyperlipidemic, gastroprotective, hepatoprotective, nephroprotective, anticancer, antifibrotic, antiproliferative, and immunostimulant. Additionally, researchers found that different parts of palm *P. dactylifera* L. have distinct beneficial chemicals. Nutraceutical substances such as anthocyanins, phenolics, sterols, carotenoids, and flavonoids have been shown to have free radical scavenging properties and to shield people from oxidative damage. Several studies have confirmed the presence of these substances in *P. dactylifera* L. palms (El-Kholy et al. 2019).

Gc-mass analysis was used to determine the phytochemical composition of ethanolic extract of *P. dactylifera* L. pits. The analysis revealed the presence of active compounds such as phenols, pentaoxacyclopen, octaethylene glycol monododecyl ether, 15,15’-Bi-1,4,7,10,13-pentaoxacyclohexadecane,á-D-Gluco pyranosiduronic acid, 5-[3,4-bis[(trimethylsilyl)oxy]phenyl]-3-methyl, and tridecane. According to El-Far et al. (2021), *P. dactylifera* L. palms have all the essential amino acids and contain phenolic and flavonoid substances, including polyphenols and flavonoids with antibacterial properties. Polyphenols can be further classified as benzoic acid derivatives and cinnamic acid derivatives. Some examples of benzoic acid derivatives are p-hydroxybenzoic acid, vanillic acid, protocatechuic acid, syringic acid, and gallic acid. On the other hand, examples of cinnamic acid derivatives are P-coumaric acid, o-coumaric acid, ferulic acid, and caffeic acid. Flavonoids, which are secondary metabolites of polyphenolic plants, are divided into many subgroups, including fava-3-ols, anthocyanidins, isoflavones, flavanones, flavanols, and flavones. Phenolic chemicals have been studied as major microbial growth inhibitors for spoilage and harmful microorganisms, particularly in the food and clinical research sectors. Phenols also have the potential to act as anti-quorum sensing agents and as inhibitors of the microbes associated with food and wound infections that form biofilms and produce toxins **(**El-Kholy et al. 2019).

*P. dactylifera* L. plum fruit is well-known for its antimicrobial properties due to its antibacterial, antifungal, and antiviral characteristics, as reported in studies by Godugu et al. (2020). In recent study, PDPE was tested against various pathogenic microorganisms, and all of them were inhibited. The highest inhibition zone diameter (IZD) of 35.0 mm was recorded for *S. aureus* ATCC 5638, while the lowest IZD was observed for *S. typhi* and *S. sonnei* with an IZD of 26.0 mm. As for the fungal strains, *A. flavus*, *P. chrysogenum*, and *A. niger*, they didn’t exhibit any zone of inhibition, but there was a failure of spores and conidia formation for all the fungi.

*P. dactylifera* L. plum fruit has been found to have antibacterial properties against a variety of bacteria such as *E. coli*, *P. aeruginosa*, *B. subtilis*, *B. cereus*, and *S. aureus* and *S. abony*, as reported in many studies including those by Samad et al. (2016). This antibacterial activity is attributed to the high phenolic content of *P. dactylifera* L. palms, which also possess antifungal properties. Previous studies have shown that *P. dactylifera* L. palm extracts have antifungal properties against several types of *A. niger, F. oxysporum*, and *C. albicans***(**El-Azim et al. 2015).

The MIC values of PDPE were tested using concentrations of 250–1000 µg/ml against the pathogenic bacteria. *S. aureus* ATCC 5638 and *E. coli* ATCC 8379 exhibited an MIC value of 500 µg/ml, whereas *B. cereus* ATCC 11778, *E. faecalis* ATCC 7080, *S. typhi* DSM 17058, and *S. sonnei* DSM 5570 exhibited an MIC value of 250 µg/ml PDPE. As investigated previously, at PDPE concentrations ranging from 500 to 1000 µg/ml the antibacterial spectrum activity showed 100% activity, whereas it was 83.3% at a concentration of 250 µg/ml. Whereas at 125 µg/ml, there was no antibacterial activity. The MBC values ranged between 500 and 1000 µg/ml of PDPE against the tested bacterial strains. *B. cereus* ATCC 11778, *E. faecalis* ATCC 7080, and *S. typhi* DSM 17058 exhibited 500 µg/ml of PDPE MBC value. *S. aureus* ATCC 5638, *S. sonnei* DSM 5570, and *E. coli* ATCC 8379 have an MBC value of 1000 µg/ml **(**Hussain et al. 2019). The antibacterial spectrum activity of PDPE at equal to 100% of 1000 µg/ml, whereas it was 66.7% at a concentration of 500 µg/ml. At 125–250 µg/ml concentrations there was no antibacterial activity against all pathogen strains. As mentioned in the previous study MIC values of *P. dactylifera* L. pits ranged from 7.80 to 4.65 mg in Ajwa and Mabroom, respectively. Hussain et al. (2019) results showed that the ethyl acetate extract of Khalas and Khodari dates inhibited *S. aureus* with an inhibition zone diameter of 20.0 mm and MIC of 10 mg/ml, while the Abu Mann pit extract both inhibited *S. aureus* and decreased the *E. coli* population. After treatment with Ajwa extracts, the inhibitory zone’s diameter was 15.0, 16.0, and 18.0 mm, and the MICs were 7.5 and 5.0 mg/ml. Different *P. dactylifera* L. pit extracts had MICs ranging from 2.5 to 10.0 mg/ml when tested against *S. aureus* ATCC 29213 and *E. coli* ATCC 25922. In the same line, Ajwa methanolic extract had antibacterial activity for *E. coli*, *B. cereus*, *S. aureus*, and *Serratia marcescens***(**Samad et al. 2016). However, Shakiba et al. (2011) stated that for the methanol extract of Mazafati dates, no inhibitory zone was seen with *E. coli* PTCC 1330, *E. coli* PTCC 1270, *E. coli* PTCC 1399, and *Serratia marcescens*. Additionally, Khatami and Shahram (2015**)** found that *P. dactylifera* L. pit aqueous extract dramatically reduced *Acinetobacter baumannii* ATCC 19606 and *Klebsiella pneumonia* PCI 602, while also decreasing the *Rhizoctonia solani* AG2_2 population at 25 µg/ml.

In a different study of Al-Daihan and Bhat (2012**)** it was found that *P. dactylifera* L. pit extract has antibacterial activity against various microbiological pathogens such as *P. aeruginosa*, *B. subtilis*, *S. aureus*, *E. coli*, *S. pyogenes*, and *S. flexeneri*. However, the aqueous extract showed little effect on *P. aeruginosa* and exhibited only a weak antibacterial effect against all tested pathogens. The study of Sadeq et al. (2021) indicated methanol and acetone pollen extracts of *P. dactylifera* pits were shown to moderately reduce the growth of both G + ve and G-ve bacteria. Another investigation carried out by Sarraf et al. (2021) using four different *P. dactylifera* L. pits revealed that ethanolic and methanolic extracts from the pits exhibited an inhibitory impact on *S. aureus* while showing no effect on *E. coli*. The extracts’ minimal inhibitory and minimal bactericidal concentrations for *S. aureus* were found to be 1.56–3.125 mg/ml and 3.125–12.5 mg/ml, respectively. The presence of active compounds such as flavanols, flavonoid glycosides, and cinnamic acids was found to be the main factor causing *P. dactylifera* L. pit antimicrobial effect **(**Zidan et al. 2023).

The PDPE biofilm inhibition showed that *S. aureus* ATCC 5638 had the highest PDPE inhibition percentage (1000 µg/ml) at 98.59%, followed by *B. cereus* ATCC 11778 at 94.12%. The lowest antibiofilm influence was observed with *E. coli* ATCC 8379 at 74.46%. In a previous study, Ajwa dates caused a significant inhibition of 70.5% and 54.19% against *Staphylococcus* sp. and *Salmonella* sp. respectively, while Safawi date has an antibiofilm effect against *Staphylococcus* sp. and *Pseudomonas* sp. with 65.78% and 45.5%, respectively. The study of Qasim et al. (2020) indicated the effective role of Ajwa and Khalas dates in preventing biofilm formation in *B. subtilis* and *Pasteurella multocida*.

Although there are limited studies on the antiviral properties of *P. dactylifera* L. palm pits, a recent investigation revealed that the highest non-toxic concentrations (CC_50_) of PDPE against Herpes simplex virus (HSV1) was 123.0 µg/ml with a 50% reduction in viral activity. Similarly, a recent study found that palm leaf extract had antiviral properties against the SARS-CoV-2 virus **(**Ahmad et al. 2022).

To conclude, *Phoenix dactylifera* L. pits ethanolic extract (PDPE) have significant antimicrobial, antibiofilm and antiviral activities. The study found that PDPE significantly influenced pathogenic microorganisms, with the most significant being *S. aureus* ATCC 5638. PDPE showed a 100% antibacterial spectrum activity against tested pathogenic bacteria, with MIC values ranging from 250 to 1000 µg/ml. *S. aureus* had the highest percentage of inhibition followed by *B. cereus* and *E. coli*. The highest non-toxic PDPE doses were found to be 123.0 µg/ml.

## Data Availability

*B. cereus* ATCC 11778 was from the ATCC collection https://www.atcc.org/products/11778. *E. faecalis* ATCC 7080 was from the ATCC collection https://www.atcc.org/products/7080. *S. aureus* ATCC 6538 was from the ATCC collection https://www.atcc.org/products/6538. *E. coli* ATCC 8379 was from ATCC collection https://www.atcc.org/products/8379 and was deposited in GenBank with taxonomy ID: NCBI: txid 481805 https://www.ncbi.nlm.nih.gov/Taxonomy/Browser/wwwtax.cgiAgNPs id = 481805. *S. typhi* DSM 17058 was from the DSM collection https://www.dsmz.de/collection/catalogue/details/culture/DSM-17058. *S. sonnei* DSM 5570 was from the DSM collection https://www.dsmz.de/collection/catalogue/details/culture/DSM-5570. *A. flavus* ATCC 9643 was from ATCC collection https://www.atcc.org/products/9643. *A. niger* was DSM 1957 from DSM collection https://www.dsmz.de/collection/catalogue/details/culture/DSM-1957. *P. chrysogenum* ATCC 10106 from ATCC collection https://www.atcc.org/products/10106.

## References

[CR1] Abd-Elhalim BT, Gamal RF, Abou-Taleb KA, Haroun AA (2019). Biosynthesis of copper nanoparticles using bacterial supernatant optimized with certain agro-industrial byproducts. J Novel Res Microb.

[CR2] Abdel-Shaheed MM, Abdalla ES, Khalil AF, El-Hadidy EM (2021). Effect of Egyptian date Palm Pollen (*Phoenix dactylifera* L.) and its hydroethanolic extracts on serum glucose and lipid profiles in Induced Diabetic rats. Food Sci Nutr.

[CR3] Ahmad M, Zain MR, Kari A, Dawood Z, Ariff MAONA, Salmuna NS, Ismail ZN, Ibrahim N, Krishnan AHT, Che Mat K, Edinur NF, Razab HAA, Mohammed MKA, Mohamed Salam A, Rao SKN, Mohamad PV, Hamat S, Zainal Abidin B, Seong Wei S, L., and, Shokri A (2022) Bioactivity and pharmacological potential of date palm (*Phoenix dactylifera* L.) against pandemic COVID-19: a Comprehensive Review. Appl Biochem Biotech 104587–4624. 10.1007/s12010-022-03952-2 10.1007/s12010-022-03952-2 PMC9110634 35579740

[CR4] Al Alawi R, Alhamdani MSS, Hoheisel JD, Baqi Y (2020). Antifibrotic and tumor microenvironment modulating effect of date palm fruit (*Phoenix dactylifera* L) extracts in pancreatic cancer. Biomed Pharm.

[CR5] Al-Daihan S, Bhat RS (2012) Antibacterial activities of extracts of leaf, fruit, seed and bark of *Phoenix dactylifera*. Afr J Biotech 11:10021–10025. 10.5897/AJB11.4309

[CR6] Aljaloud S, Colleran HL, Ibrahim SA (2020) Nutritional value of date fruits and potential use in nutritional bars for athletes. Food Sci Nutr 11(06):463. 10.4236/fns.2020.116034

[CR7] Ansari MA, Khan HM, Khan AA, Swaranjit SC, Pal R (2014) Antibiofilm efficacy of silver nanoparticles against biofilm of extended-spectrum β-lactamase isolates of *Escherichia coli* and *Klebsiella pneumoniae*. Appl Nanosci 4:859–868. 10.1007/s13204-013-0266-1

[CR8] Bouhlali T,; Alem G, Ennassir J, Benlya M, Mbark AN, Zegzouti YF (2015) Phytochemical compositions and antioxidant capacity of three date (*Phoenix dactylifera* L.) seeds varieties grown in the South East. J. Saudi Soc. Agric. Sci. 2017, 16, 4, 350 – 35. 10.1016/j.jssas.2015.11.002

[CR9] Daoud A, Malika D, Bakari S, Hfaiedh N, Mnafgui K, Kadri A, Gharsallah N (2019). Assessment of polyphenol composition, antioxidant and antimicrobial properties of various extracts of date palm pollen (PDPE) from two Tunisian cultivars. Arab J Chem.

[CR10] Duncan DB (1955). Multiple range and multiple F test. Biomet.

[CR12] El-Azim MHMA, Yassin FA, Khalil SA, El-mesalamy AMD (2015). Hydrocarbons, fatty acids, and biological activity of date palm pollen (*Phoenix dactylifera* L.) growing in Egypt. IOSR J Pharm Biol Sci Ver I.

[CR13] El-Far AH, Rokaia F, Ragab, Shaker A Mousa. Date Palm Bioactive compounds: nutraceuticals, functional nutrients, and Pharmaceuticals. Date Palm Genome (2021) 2 Mol B, 27–50. 10.1007/978-3-030-73750-4_2

[CR14] El-Kholy WM, Tarek N, Soliman, Amira M, Galal Darwish (2019). Evaluation of date Palm Pollen (*Phoenix Dactylifera* L.) Encapsulation, Impact on the Nutritional and Functional properties of fortified Yoghurt. PLoS ONE.

[CR11] Elakraa AA, Salem S, Salem, Gharieb S, El-Sayyad, Mohamed S (2022). Attia. Cefotaxime Incorporated Bimetallic Silver-Selenium nanoparticles: promising Antimicrobial synergism, Antibiofilm Activity, and bacterial membrane leakage reaction mechanism. RSC Advan.

[CR15] Franchi G, Bovalini L, Martelli P, Ferri S, Sbardellati E (1985). High-performance liquid chromatography analysis of the *furanochromones khellin* and visnagin in various organs of *Ammi visnaga* (L.) Lam at different developmental stages. J Ethno Pharmacol.

[CR16] Galal, Gehan F, Basma T, Abd-Elhalim KA, Abou-Taleb AA, Haroun, Rawia F, Gamal (2021) Toxicity assessment of green synthesized Cu nanoparticles by cell-free extract of *Pseudomonas silesiensis* as antitumor cancer and antimicrobial. Ann Agric Sci 66(1):8–15. 10.1016/j.aoas.2021.01.006

[CR17] Godugu K, El-Far AH, Jaouni SA (2020). Mousa. Nano formulated Ajwa (*Phoenix Dactylifera*) Bioactive compounds improve the safety of Doxorubicin without compromising its Anticancer efficacy in breast Cancer. Mol.

[CR18] Gregorova M, Morse D, Brignoli T, Steventon J, Hamilton F, Albur M, Arnold D, Thomas M, Halliday A, Baum H, Rice C, Avison MB, Davidson AD, Santopaolo M, Oliver E, Goenka A, Finn A, Wooldridge L, Amulic B, Massey RC (2020) Post-acute COVID-19 associated with evidence of bystander T-cell activation and a recurring antibiotic-resistant bacterial pneumonia. ELife 9. 10.7554/eLife.63430 10.7554/eLife.63430 PMC7775105 33331820

[CR19] Humphries RM, Ambler J, Mitchell SL, Castanheira M, Dingle T, Hendler JA, Koeth L, Sei K, CLSI Methods Development and Standardization Working Group of the Subcommittee on Antimicrobial Susceptibility Testing (2018) CLSI Methods Development and Standardization Working Group Best Practices for Evaluation of Antimicrobial susceptibility tests. J Clin Microb 56(4). 10.1128/jcm01934-17 10.1128/JCM.01934-17 PMC5869819 29367292

[CR20] Hussain MI, Mohammad H, Semreen A, Shanableh, Muhammad N, Khan Khattak I, Saadoun IM, Ahmady M, Mousa N, Darwish W, Radeef, Sameh SM (2019). Soliman. Phenolic composition and antimicrobial activity of different Emirati date (*Phoenix Dactylifera* L.) pits: a comparative study. Plants.

[CR21] Khatami M, Shahram P (2015) *Phoenix dactylifera* (date palm) pit aqueous extract mediated novel route for synthesis high stable silver nanoparticles with high antifungal and antibacterial activity. IET Nanobiotech 9:184–190. 10.1049/iet-nbt.2014.0052 10.1049/iet-nbt.2014.0052 26224347

[CR22] Mia MA-T, Mosaib MG, Khalil MI, Islam MA, Gan SH (2020). Potentials and safety of date palm fruit against diabetes: a critical review. Foods.

[CR23] Mirza MB, Elkady AI, Al-Attar AM, Syed FQ, Mohammed FA, Hakeem KR (2018). Induction of apoptosis and cell cycle arrest by ethyl acetate fraction of *Phoenix dactylifera* L. (Ajwa dates) in prostate cancer cells. J Ethnopharma.

[CR24] Petricevich VL, Mendonça RZ (2003). Inhibitory potential of *Crotalus durissus terrificus* venom on measles virus growth. Toxicon: Official J Int Soci Toxino.

[CR25] Platat C, Hillary S, Tomas-Barberan FA, Martinez-Blazquez JA, Al-Meqbali F, Souka U, Al-Hammadi S, Ibrahim W (2019). Urine metabolites and antioxidant effect after oral intake of date (*Phoenix dactylifera* L.) seeds-based products (powder, Bread and Extract) by human. Nutrie.

[CR26] Qasim N, Shahid M, Yousaf F, Riaz M, Anjum F, Faryad MA, Shabbir R (2020). Therapeutic potential of selected varieties of *Phoenix dactylifera* L. Against microbial biofilm and free radical damage to DNA. DOS.

[CR27] Reed JC (1999). Dysregulation of apoptosis in cancer. J Clin Oncol.

[CR28] Reed LJ, Muench H (1938). A simple method of estimating 50% endpoints. Am J Hyg.

[CR29] Sadeq O, Mechchate H, Es-Safi I, Bouhrim M, Jawhari FZ, Ouassou H, Kharchoufa L, N AlZain M M, Alzamel N, Mohamed Al Kamaly O, Bouyahya A, Benoutman A, Imtara H (2021) 1;10(4):676 Phytochemical Screening, Antioxidant and Antibacterial Activities of Pollen Extracts from *Micromeria fruticosa*, *Achillea fragrantissima*, and *Phoenix dactylifera*. Plants (Basel). 10.3390/plants10040676 10.3390/plants10040676 PMC8066694 33915923

[CR30] Samad MA, Hashim SH, Simarani K, Yaacob JS (2016). Antibacterial properties and effects of fruit chilling and extract storage on antioxidant activity, total phenolic and anthocyanin content of four date palm *(Phoenix dactylifera*) cultivars. Mol.

[CR31] Sarraf M, Jemni M, Kahramanoğlu I, Artés F, Shahkoomahally S, Namsi A, Ihtisham M, Brestic M (2021). Mostafa Mohammadi, and Anshu Rastogi. Commercial techniques for preserving date palm (*Phoenix Dactylifera*) Fruit Quality and Safety: a review. Saudi J Biol Sci.

[CR32] Saryono S, Taufik A, Proverawati A, Efendi F (2019). Dietary supplementation of *Phoenix dactylifera* L. seeds decreases pro-inflammatory mediators in CCl4-induced rats. J Herb Med Pharma.

[CR33] Shakiba M, Kariminik A, Parsia P (2011) Antimicrobial activity of different parts of *Phoenix dactylifera*. Int J Mol Clin Microb 1:107–111

[CR34] Shanableh A, Radeef W (2020). Biogas production from raw and oil-spent date palm seeds mixed with wastewater treatment sludge. Biofuels.

[CR35] Zidan N, Albalawi MA, Adel I, Alalawy MA, Al-Duais S, Alzahrani M, Kasem AA, Tayel, Rasha M (2023). Nagib. Active and smart antimicrobial Food Packaging Film composed of date palm kernels extract loaded Carboxymethyl Chitosan and Carboxymethyl Starch Composite for prohibiting foodborne pathogens during fruits preservation. Eur Polym J.

[CR36] Zihad NK, Uddin SJ, Sifat N, Lovely F, Rouf R, Shilpi JA, Sheikh BY, Göransson U (2021). Antioxidant properties and phenolic profiling by UPLC-QTOF-MS of Ajwah, Safawy, and Sukkari cultivars of date palm. Biochem Biophys Rep.

